# Association of resident training with complication risk in total hip and knee arthroplasty: a systematic review and meta-analysis

**DOI:** 10.2340/17453674.2025.43905

**Published:** 2025-11-17

**Authors:** Diederik H R KEMPEN, Barry VAN DER ENDE, Diyar DELAWI, Chantal DEN HAAN, Amy HOFMAN, Rudolf W POOLMAN, Nienke WOLTERBEEK

**Affiliations:** 1 Department of Orthopedic Surgery, Joint Research, OLVG Amsterdam, Amsterdam; 2 Department of Orthopedic Surgery, Amsterdam University Medical Center, Amsterdam; 3 Department of Orthopedic Surgery, St. Antonius Hospital, Utrecht; 4 Department of Research and Epidemiology, OLVG Amsterdam, Amsterdam; 5 Department of Orthopaedic Surgery, Leiden University Medical Center, Leiden, The Netherlands

## Abstract

**Background and purpose:**

An important aspect of orthopedic residency is to gain experience and perform total joint arthroplasties to learn operating skills. For patients, a substantial concern is that resident involvement may result in more adverse events. We performed a systematic review with meta-analysis to evaluate whether resident involvement is associated with a higher complication rate in total hip and knee arthroplasty compared with procedures performed by orthopedic surgeons.

**Methods:**

PubMed, Embase, Central, Cinahl, and Web of Science were systematically searched until November 2, 2023. Studies were assessed by 2 reviewers independently. All manuscripts were included describing: (i) complications in total hip and/or knee arthroplasty and (ii) comparing procedures with and without resident involvement.

**Results:**

29 studies could be included with a MINORS score ranging from 10 to 17. Resident involvement could not be associated with an increased overall and surgical complication rate in 9 (n = 51,442 patients) and 12 studies (n = 37,789 patients), respectively. The meta-analysis for deep infection showed an association with resident involvement (RR 1.55, 95% confidence interval 1.09–2.20, P = 0.01, 18 studies). In single studies, associations were seen for any urologic complication and sepsis. All other complications could not be related to resident involvement.

**Conclusion:**

This systematic review could not find evidence that resident involvement was associated with more overall complications or surgical site complications except for deep infection. As previous studies showed an increased infection risk with prolonged duration of the procedure, consultants in teaching hospitals should be aware of this risk and be alert to teaching time to limit the risk for patients.

The goal of orthopedic surgery training programs is to provide operative training for young physicians while continuing safe and efficacious patient care. Training programs have evolved making sure that trainees (residents) are gradually exposed to surgical procedures with increasing complexity. During training, surgical skills are acquired predominantly in the operating room where the resident is supervised by an experienced orthopedic surgeon. Because both the surgeon and resident actively contribute to the operation, every step of the procedure receives attention. Although surgical training is an important part of the residency program, patients may prefer a surgeon with more experience performing the procedure. One of the reasons is the societal concern that resident involvement may result in more adverse events. Our systematic review aims to investigate whether resident involvement is associated with a higher complication rate in total hip and knee arthroplasty (THA/TKA) compared with procedures performed by orthopedic surgeons.

## Methods

The systematic literature review regarding the association of resident involvement with complications after THA and TKA was reported according to the Preferred Reporting Items for Systematic reviews and Meta-Analyses (PRISMA) guidelines [1]. We defined the following PICO question: our population (P) as patients who underwent THA or TKA [2]. The intervention (I) was defined as orthopedic residents involved in the surgical procedure. We compared (C) this intervention with procedures performed by orthopedic surgeons for the outcome (O) all reported complications. Study design (S) consisted of all published literature with the exception of reviews.

### Literature search

The protocol was not registered in an international database, which is why we were unable to comply with PRISMA item 24a.

A search strategy was designed supported by a specialist (CdH), to identify relevant publications in PubMed, Embase. com, CENTRAL, Cinahl/Ebscohost, and Web of Science. The search included index terms as well as free text words. Supplementary Table 1 summarizes the full search strategy for all databases. The electronic search was updated on November 2, 2023, searching all databases from inception. Literature in English, German, French, and Dutch was included.

### Study selection and data extraction

All manuscripts were included that described: (i) complications in primary THA and/or TKA and (ii) comparing procedures with and without resident involvement. Studies that did not differentiate between procedures with and without resident involvement were excluded. 2 reviewers (DK and NW) independently assessed the literature by screening title and abstract using Rayyan [3]. Subsequently, full-text articles were examined for eligibility. In addition, bibliographies of all included full-text articles were reviewed to identify potential additional eligible articles. During the selection, the reviewers were blinded to each other’s assessment.

Data extraction of the selected manuscripts was performed independently by 2 reviewers (DK and NW) using a predefined data extraction sheet. The original manuscripts were reviewed without blinding for authors and affiliation. Relevant data regarding the country of origin, study type, data source, joint(s) involved, number of patients, follow-up, mortality, and any reported complication was extracted from the text and tables. All complications reported in the study were extracted from the studies. Studies describing summarized complication rates were included in the analysis of the overall and surgical site complication analysis.

Study quality was determined independently by the 2 reviewers using a standardized grading tool (MINORS criteria) [4]. The items were scored 0 if not reported, 1 when reported but inadequate and 2 when reported and adequate. For comparative studies, the scores can be interpreted as 0–6, very low quality; 7–12, low quality; 13–18 fair quality; and 19–24, high quality [5]. Disagreements between reviewers in manuscript selection, data extraction, and grading of study quality were resolved in a consensus meeting. To assess the risk of bias due to missing evidence according to the RoB-ME tool, a cross-check of the methods and results section was done one against the other to identify any outcomes with no or incomplete results reported.

### Statistics

A meta-analysis of the studies was performed using Review Manager software (RevMan Version 5.3, Copenhagen: The Nordic Cochrane Centre, The Cochrane Collaboration, 2008). Risk ratios were calculated to quantify how strongly the mortality and complications were associated with the involvement of residents. For combining the results found in the different trials the statistical method of Mantel–Haenszel with random effects method was used for dichotomous outcomes, and risk ratios for THA and TKA procedures with and without resident involvement were calculated. Heterogeneity between studies was assessed by using I^2^ statistics. I^2^ values between 30–60%, 50–90%, and 75–100% represent moderate, substantial, and considerable heterogeneity, respectively [6]. Risk ratios (RR) with 95% confidence intervals (CI) were reported and a P value of < 0.05 was considered statistically significant.

### Ethics, data sharing plan, funding, use of AI, and disclosures

For this systematic review, no ethical approval was required. For data-sharing purposes, extracted data is provided in the supplementary files. During this project and the preparation of the manuscript, no artificial intelligence tools were used. No funding was received for this study. The authors declare that they have no conflicts of interest regarding this research. Complete disclosure of interest forms according to ICMJE are available on the article page, doi: 10.2340/17453674.2025.43905

## Results

### Study selection and data extraction

The search strategy yielded 4,431 articles, of which 2,491 remained after deduplication (Figure). Of these studies, 2,366 were excluded after screening of titles and abstracts. After fulltext screening of the remaining 125 articles, 29 articles were included for this review. All excluded articles did not provide data describing complications in populations operated on with or without resident involvement. Cross-referencing did not result in additional studies. 2 prospective and 27 retrospective cohort studies [7-35] were included consisting of 12 examining complications in THA, 12 in TKA, and 5 in both (Table 1). The number of patients ranged from 50 to 89,087 and followup duration between discharge from the ward and 10 years. For the assessment of the risk of bias due to missing evidence no study protocols or pre-specified analysis plans were found for the cohort studies. Although reporting of complications in the methods section was often limited, reporting of the outcome was detailed, and no discrepancies were found when cross-checking the methods and results section. Due to the poor description of complications in the methods section and nature of the studies, selective reporting bias in some studies is likely. The direction of the possible bias is unpredictable. The reporting on outcomes in the methods section and the extracted complications from each study are provided in Supplementary Table S2.

**Figure:**
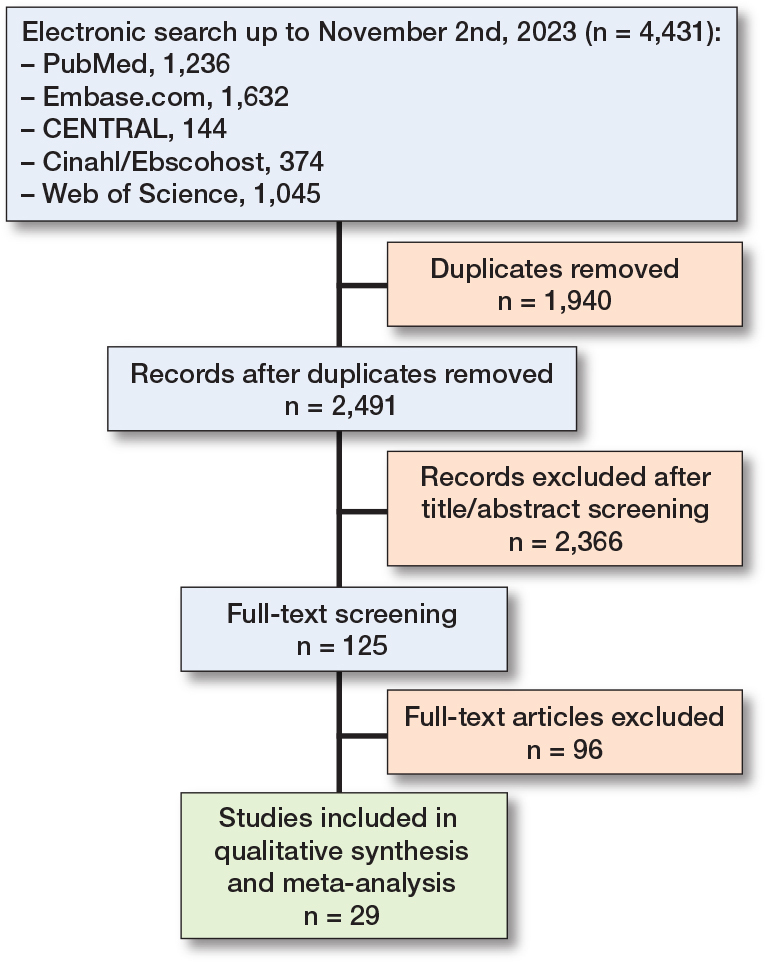
Flowchart of study.

**Table 1:** Overview of studies

1st author	Country	Study type ^a^	Data source ^b^	Joint ^c^	Total	Patients, n Residents	OS	Follow-up	Ref
Lederer 2001	Germany	R	Single center	h	3,290	1,290	2,000	ND	7
Moran 2004	UK	P	Single center	h	536	139	397	1.5 years	8
Robinson 2007	USA	R	Single center	h	135	52	83	1 year	9
Woolson 2007	USA	R	Single center	h & k	401	191	210	> 6 months	10
Palan 2009	UK	R	Multi-center	h	1,501	528	973	5 years	11
Inglis 2012	New Zealand	R	NZJR 2005-2011	h	34,393	4,049	30,344	6 years	12
Schoenfeld 2013	USA	R	NSQIP 2005-2010	h & k	23,783	4,816	18,967	30 days	13
Bohl 2014	USA	R	NSQIP 2006-2010	h & k	21,434	4,260	17,174	30 days	14
Haughom 2014	USA	R	NSQIP 2005-2012	h	13,109	3,462	9,647	ND	15
Haughom 2014	USA	R	NSQIP 2005-2012	k	24,529	5,960	18,569	ND	16
Hasegawa 2015	Japan	R	Single center	h	483	259	224	5.1 years	17
Reidy 2016	UK	R	Multi-center	h	870	286	584	10 years	18
Wilson 2016	Australia	R	Single center	h & k	4918	2,313	2,605	ND	19
Faulkner 2017	UK	R	Multi-center	k	686	236	450	10 years	20
Weber 2017	Germany	R	Single center	h	1,008	240	768	1 year	21
Weber 2017	Germany	R	Single center	k	738	292	446	1 year	22
Windisch 2017	Germany	R	Single center	k	1,068	80	988	3 years	23
Beattie 2018	UK	R	Single center	k	609	144	465	1 year	24
Smith 2018	New Zealand	R	NZJR 2000-2014	h	89,087	9,586	79,501	0.5 year	25
Theelen 2018	Netherlands	R	Single center	k	642	220	422	4.5 years	26
MacDonald 2019	UK	R	Single center	h	87	44	43	13 months	27
Nakamura 2019	Japan	P	Single center	k	100	58	42	> 3 months	28
Foissey 2020	France	R	Single center	h	488	147	341	3 years	29
Bron 2021	Netherlands	R	Multi-center	h & k	7,123	2,027	5,096	> 3 months	30
Hoerlesberger 2021	Austria	R	Single center	k	206	103	103	Till discharge	31
Sheridan 2022 **^d^**	Canada	R	Single center	k	315	315	315	>4 years	32
Maheshwari 2022 **^d^**	USA	R	Single center	k	50	50	50	1 year	33
Anis 2022	USA	R	Multi-center	k	12,664	7,234	5,430	1 year	34
Stafford 2023	USA	R	Single center	k	200	100	100	14 months	35

a Study type: prospective (P) or retrospective (R) cohort study.

b Data source: New Zealand Joint Registry (NZJR) and National Surgical Quality Improvement Program (NSQIP).

c h: hip; k: knee.

d Bilateral knee replacement (one side consultant, other side resident).

ND: no data; OS: orthopedic surgeon.

The mean MINORS score was 13.7 ranging from 10 to 17 (Table 2). The quality can be interpreted as low in 9 studies and fair in 20 studies. 2 studies used the New Zealand Joint Registry (NZJR) and 4 studies the National Surgical Quality Improvement Program (NSQIP) as the source of their data [12-16,25]. To avoid including overlapping cohorts for some complications, a selection of these studies was made during data analysis. The studies with the highest number of reported complications for hip and knee arthroplasties combined were included in the analysis.

**Table 2:** Quality of the included studies

Study	A	B	C	D	E	F	G	H	I	J	K	L	M
Lederer 2001	2	2	0	2	0	0	0	0	2	2	0	1	11
Moran 2004	1	0	2	1	0	2	1	0	2	2	1	1	13
Robinson 2007	2	0	1	1	0	2	1	1	2	2	0	1	13
Woolson 2007	1	2	1	2	0	2	0	2	2	2	0	1	15
Palan 2009	2	0	2	1	0	2	1	0	2	2	0	1	13
Inglis 2012	1	0	0	1	0	2	0	0	2	2	0	2	10
Schoenfeld 2013	2	0	0	2	0	2	0	0	2	2	0	2	12
Bohl 2014	1	0	0	2	0	1	0	0	2	2	0	2	10
Haughom 2014	2	0	0	2	0	1	0	0	2	2	1	2	12
Haughom 2014	2	0	0	2	0	1	0	0	2	2	1	2	12
Hasegawa 2015	1	2	2	2	0	2	1	0	2	2	0	1	15
Reidy 2016	2	2	0	1	0	2	2	0	2	2	0	2	15
Wilson 2016	1	2	2	1	0	2	0	0	2	2	1	1	14
Faulkner 2017	2	2	1	2	0	2	1	0	2	2	1	2	17
Weber 2017	2	1	1	2	0	2	0	2	2	2	1	1	16
Weber 2017	2	1	1	2	0	2	0	2	2	2	1	1	16
Windisch 2017	2	1	1	2	0	2	0	0	2	2	0	1	13
Beattie 2018	2	2	1	1	0	2	0	0	2	2	2	1	15
Smith 2018	2	0	0	1	0	2	0	0	2	2	1	1	11
Theelen 2018	2	1	0	1	0	2	0	0	2	2	2	1	13
MacDonald 2019	2	2	1	2	0	1	0	0	2	2	0	0	12
Nakamura 2019	1	2	2	1	0	1	2	0	2	2	2	1	16
Foissey 2020	2	1	1	1	0	2	2	0	2	2	1	1	15
Bron 2021	2	2	1	2	0	2	0	0	2	2	1	2	16
Hoerlesberger 2021	2	1	0	1	0	0	0	2	2	2	1	1	12
Sheridan 2022	2	1	0	1	0	2	1	0	2	2	2	2	15
Maheshwari 2022	1	1	0	1	1	1	2	0	2	2	2	2	15
Anis 2022	2	2	0	1	0	1	2	0	2	2	1	2	15
Stafford 2023	2	2	0	2	0	1	2	0	2	1	2	1	15

A Clearly stated aim

B Inclusion of consecutive patients

C Prospective collection of data

D Endpoint appropriate to study aim

E Unbiased assessment of endpoints

F Follow-up appropriate to study aim

G < 5% loss to follow-up

H Prospective calculation of study size

I Adequate control group

J Contemporary groups

K Baseline equivalance of groups

L Adequate statistical analysis

M Total

### Overall complication rate and mortality

9 (n = 51,442) and 12 (n = 37,789) studies were included in the analysis of the overall and surgical complication rate, respectively. Although there was substantial heterogeneity, pooled data showed that resident involvement was not significantly associated with a higher overall or surgical complication rate compared with procedures performed by an orthopedic surgeon (Table 3 and Supplementary Forest plots S3 A–B). Comparison of the mortality was performed in 6 studies (n = 32,768) and showed no significant association of the involvement of residents with early death (< 90 days) (Supplementary Forest plot S3 C).

**Table 3:** Overall risk ratios for complications

Complication	Studies (n)	Residents (n/N)	Orthopedic surgeon (n/N)	RR (CI)	P value	I^2^ (%)
Overall complications	9	1,549/14,428	3,007/37,014	1.07 (0.97-1.19)	0.2	46
Surgical complication	12	514/9,860	1,232/27,929	1.04 (0.88-1.24)	0.6	39
Mortality	6	24/7,485	66/25,283	1.27 (0.79-2.04)	0.3	0

CI: 95% confidence interval.

See supplementary forest plot files for details.

### Surgical site complications

Of the investigated complications, deep infection could be associated with resident involvement (RR 1.55, CI 1.09–2.20): 193 of 27,170 (0.7%) of the cases with a resident involved developed a deep infection compared with 342 of 116,053 (0.3%) cases without resident involvement (Table 4 and Supplementary Forest plot S4A). Although there was heterogeneity for some of the complications, none of the other surgical complications (reoperation, THA dislocation, fracture, nerve palsy, superficial infection, aseptic loosening, limited mobility of TKAs, instability of TKAs, wound defect, malalignment of TKAs, serious drainage/hematoma, wound complications [not specified], acetabular perforations, blistering, patellar dislocation in TKAs, psoas pain/impingement, arterial injury subluxations, insert wear in TKAs, patellar wear, heterotopic ossifications, and patellar clunk) could be associated with resident involvement (Table 4 and Supplementary Forest plots S4 B–L). Pooling of the data on aseptic loosening bordered the significance level set for this meta-analysis (P = 0.08, Supplementary Forest plot S4 G).

**Table 4:** Risk ratios of surgical site complications

Complication	Studies (n)	Residents (n/N)	Orthopedic surgeon (n/N)	RR (CI)	P value	I^2^ (%)
Overall complications	9	1,549/14,428	3,007/37,014	1.07 (0.97-1.19)	0.2	46
Deep infection	18	193/27,170	342/116,053	1.55 (1.09-2.20)	0.01	48
Reoperation	14	269/17,684	890/68,379	1.11 (0.92-1.34)	0.3	17
Dislocation THA	12	113/8,304	304/38,746	1.17 (0.94-1.46)	0.2	0
Fracture	9	33/6,843	119/35,180	0.90 (0.59-1.37)	0.6	0
Nerve palsy	9	64/9,222	102/27,690	1.42 (0.83-2.43)	0.2	39
Superficial infection	7	264/17,244	376/35,589	0.99 (0.84-1.17)	0.9	0
Aseptic loosening	7	24/5,209	95/32,372	1.50 (0.95-2.37)	0.08	0
Limited mobility TKA	5	17/893	14/1,336	1.60 (0.80-3.21)	0.2	0
Instability TKA	2	2/316	5/1,438	2.18 (0.08-58.95)	0.6	72
Wound defect	4	23/9,901	56/28,862	1.21 (0.74-1.98)	0.5	0
Malalignment TKA	2	0/308	4/549	0.32 (0.04-2.74)	0.3	0
Serious drainage/hematoma	2	5/243	3/293	1.81 (0.47-6.94)	0.4	0
Wound complication **^a^**	1	160/2,313	175/2,605	1.03 (0.84-1.27)	0.8	NA
Acetabular perforations	1	9/1,290	10/2,000	1.40 (0.57-3.42)	0.5	NA
Blistering	1	0/220	3/422	0.27 (0.01-5.27)	0.4	NA
Patellar dislocation TKA	1	0/220	2/422	0.38 (0.02-7.94)	0.5	NA
Psoas pain/impingement	1	4/147	18/341	0.52 (0.18-1.50)	0.2	NA
Gluteus tendinitis	1	2/147	8/341	0.58 (0.12-2.70)	0.5	NA
Arterial injury	1	2/259	0/224	4.33 (0.21-90)	0.3	NA
Subluxations	1	4/191	1/210	4.40 (0.50-39)	0.2	NA
Insert wear TKA	1	0/191	1/210	0.37 (0.02-8.94)	0.5	NA
Patellar wear	1	1/191	1/210	1.10 (0.07-17)	0.9	NA
Heterotopic ossifications	1	3/119	0/111	6.53 (0.34-125)	0.2	NA
Patellar clunk	1	0/50	1/50	0.33 (0.01-7.99)	0.5	NA

CI: 95% confidence interval; TKA: total knee arthroplasty; NA: not applicable.

See supplementary forest plot files for details.

a Unspecified.

### Systemic complications

None of the thrombo-embolic, pulmonary, cardiac, neurological, or gastrointestinal complications were associated with involvement of residents during total knee and/or hip arthroplasty. Only any urological complications (RR 1.52, CI 1.17–1.96) and sepsis (RR 1.80 CI 1.22–2.65) happened significantly more when a resident was involved (Table 5 and Supplementary Forest plots S5 A–L).

**Table 5:** Risk ratios of systemic complications

Complication	Studies (n)	Residents (n/N)	Orthopedic surgeon (n/N)	RR (CI)	I^2^ P value	(%)
Pulmonary ^a^						
On ventilator > 48 h	2	12/9,422	20/28,216	1.79 (0.88-3.64)	0.1	37
Unplanned intubation	2	19/9,422	59/28,216	0.96 (0.57-1.61)	0.9	42
Pneumonia	2	35/9,422	100/28,216	0.88 (0.29-2.70)	0.8	83
Any pulmonary compl.	1	14/2,027	34/5,096	1.04 (0.56-1.92)	0.9	NA
Cardiac						
Cardiac arrest	2	6/9,422	27/28,216	0.70 (0.29-1.71)	0.4	0
Myocardial infarction	2	29/9,422	61/28,216	1.44 (0.92-2.23)	0.1	0
Any cardiac compl.	1	35/2,027	68/5,096	1.29 (0.86-1.94)	0.2	NA
Hypotension	1	3/220	1/422	5.75 (0.60-55)	0.1	NA
Hypovolemic shock	1	1/220	0/422	5.74 (0.23-140)	0.3	NA
Cardiac arrythmia	1	1/220	0/422	5.74 (0.23-140)	0.3	NA
Thrombo-embolic						
Deep venous thrombosis	6	126/11,106	289/30,795	1.17 (0.95-1.44)	0.2	0
Pulmonary embolism	6	74/11,220	169/31,171	1.21 (0.87-1.68)	0.3	10
Neurological						
Cerebrovascular accident	3	14/11,449	48/33,312	0.85 (0.46-1.54)	0.6	0
Delirium	1	41/2,027	78/5,096	1.32 (0.91-1.92)	0.1	NA
Coma	1	1/4,260	0/17,174	12.1 (0.49-296)	0.1	NA
Renal/Urological						
Renal insufficiency	2	8/,422	37/28,216	0.66 (0.31-1.43)	0.3	0
Acute renal failure	2	5/9,422	29/28,216	0.52 (0.20-1.35)	0.2	0
Urinary tract infection	2	144/9,422	401/28,216	1.07 (0.89-1.30)	0.5	0
Any urologic compl.	1	90/2,027	149/5,096	1.52 (1.17-1.96)	0.001	NA
Gastrointestinal	1	10/2,027	21/5,096	1.20 (0.56-2.54)	0.6	NA
Infectious						
Sepsis	1	37/4,816	81/18,967	1.80 (1.22-2.65)	0.003	NA
Septic shock	1	4/4,816	29/18,967	0.54 (0.19-1.54)	0.3	NA
Organ infection	1	7/4,260	23/17,174	1.23 (0.53-2.86)	0.6	NA
Unplanned readmission	2	92/9,422	230/28,216	1.20 (0.94-1.53)	0.1	NA

a Pulmonary embolism excluded. NA: not applicable. See supplementary forest plot files for details.

## Discussion

We aimed to investigate the association between resident involvement and complication rate in total hip and knee arthroplasty and showed no significant association with overall complications, overall surgical complications, or mortality. For each of the individually reported surgical complications, a significant association was found only for resident involvement and deep infection. For the individual systemic complications, resident involvement was associated with a significant difference for any urologic complications and sepsis but only based on 2 studies [13,30]. Overall, these results imply that our current way of training orthopedic surgeon may expose patients to a slight increased infection risk for complications during total joint arthroplasty.

In contrast to previous reviews, this is the first report finding an association between resident involvement and deep infection [36-38]. The increased infection risk may be explained by increased surgical time in trainee involved procedures [9,11,21,34,37-39]. Increased surgical time has proven in multiple studies to be an independent risk factor for infection in total joint arthroplasty [40-43]. In a recent study, resident involvement did not independently increase the risk for infection. However, cases with resident involvement were associated with longer operative times and patients with increased medical complexity, both of which were found to be independently associated with an increased risk of infection [34]. It could very well be that there is a logical longer duration of the procedure due to “teaching time,” which may have increased infection risk [44-47]. However, this does not result in a higher number of other complications.

### Limitations

Due to the nature of the retrospective cohort studies, non-reporting of risk factors and reporting bias are likely present in some studies. Furthermore, selection bias will also be likely in these studies as complex patients with higher infection risks are more often treated in large teaching hospitals instead of smaller private clinics without resident training programs. Low surgeon volume and prolonged duration of the surgical procedure are also mentioned as risk factors. Naturally, the exposure of residents to high volumes of total joint arthroplasty is still limited during their training. Another limitation could be the heterogeneity within training programs because trainees receive different kinds of responsibilities and independence in the operating theatre according to their progression and number of years in training.

### Clinical implications

Although there are no guidelines stating which differences in risk ratio for complications are acceptable or clinically important, deep infection is one of the most serious complications in total joint arthroplasty and the risk should be minimized. We suggest that training programs should be alert to time, so that thresholds for increased infection risks are not overrun. Each procedure should be divided into different parts or steps and given a maximum duration. If the trainee exceeds this time, the consultant or supervisor should take over. These parts of the surgery can also be allocated to trainee or consultant before the start of the procedure to put more focus on the learning objective for the specific procedure.

### Conclusion

We showed no evidence of an association between resident involvement and more overall or surgical site complications in primary TKAs and THAs compared with orthopedic surgeons. However, resident involvement was associated with a higher risk of deep infection, any urologic complications, and sepsis.

*In perspective*, it is recommended to be alert to the training time and maximum duration of the surgical procedure. This can help teaching hospitals to improve the resident training program and may help to reassure patients.

## Supplementary Material



## Supplementary data

Search terms, reported complications per article, and Tables with forest plots of complications are available as Supplementary data on the article page, doi: 10.2340/17453674.2025.43905

All co-authors (DK, BE, DD, CdH, AH, RP, NW) contributed to the concept and design of the study. CdH performed the literature search, DK and NW performed the study selection and data extraction. DK, BE, DD, and NW contributed to the writing process. All authors revised and approved the manuscript.

Handling co-editors: Marianne Westberg and Robin Christensen

*Acta* thanks Volker Thomas Christian Otten and Christof Wagner for help with peer review of this manuscript.
