# Supplementary material for: Association of resident training with complication risk in total hip and knee arthroplasty: a systematic review and meta-analysis
Source: Acta Orthop. 2025 Nov 17;96:850–6. doi: 10.2340/17453674.2025.43905 (PMC12621420; doi:10.2340/17453674.2025.43905)
Supplement: Supplementary file 1 [file ActaO-96-43905-s1.pdf]

Supplementary Table S1. Search terms

Database	Search term		
**Pubmed**	(("Education, Medical, Graduate"[Mesh] OR (house[tiab] AND officer*[tiab]) OR resident*[tiab] OR residency[tiab] OR trainee*[tiab] OR registrar*[tiab] OR education[sh] OR (junior[tiab] AND surgeon*[tiab]))) AND (("Arthroplasty, Replacement, Knee"[Mesh] OR "Arthroplasty, Replacement, Hip"[Mesh] OR "Knee Prosthesis"[Mesh] OR "Hip Prosthesis"[Mesh] OR TKA[tiab] OR TKR[tiab] OR THA[tiab] OR THR[tiab] OR total hip*[tiab] OR total knee*[tiab] OR (("Knee Joint"[Mesh] OR "Knee"[Mesh] OR "Hip"[Mesh] OR "Hip Joint"[Mesh] OR "Joints"[Mesh] OR knee[tiab] OR knees[tiab] OR joint[tiab] OR hip[tiab] OR hips[tiab] OR femur*[tiab] OR femoral[tiab]) AND ("Arthroplasty, Replacement"[Mesh] OR replacement*[tiab] OR arthroplast*[tiab] OR artroplast*[tiab] OR prosthes*[tiab] OR prosthet*[tiab] OR alloplast*[tiab] OR implant*[tiab]))))		1236
**Embase**	('graduate medical education'/exp OR 'resident'/exp OR 'residency education'/exp OR 'surgical training'/exp OR ((house NEAR/2 officer*):ab,ti) OR resident*:ab,ti OR residency:ab,ti OR trainee*:ab,ti OR registrar*:ab,ti OR ((junior NEAR/2 surgeon*):ab,ti)) AND ('knee replacement'/exp OR 'hip replacement'/exp OR 'knee prosthesis'/exp OR 'hip prosthesis'/exp OR tka:ab,ti OR tkr:ab,ti OR tha:ab,ti OR thr:ab,ti OR 'total hip*':ab,ti OR 'total knee*':ab,ti OR (('knee'/exp OR 'hip'/exp OR 'joint'/exp OR knee:ab,ti OR knees:ab,ti OR joint:ab,ti OR hip:ab,ti OR hips:ab,ti OR femur*:ab,ti OR femoral:ab,ti) AND ('replacement arthroplasty'/exp OR replacement*:ab,ti OR arthroplast*:ab,ti OR artroplast*:ab,ti OR prosthes*:ab,ti OR prosthet*:ab,ti OR alloplast*:ab,ti OR implant*:ab,ti)))		1632
**CENTRAL**	#1	((house NEAR/2 officer*) OR resident* OR residency OR trainee* OR registrar* OR (junior NEAR/2 surgeon*)):ti,ab,kw	20407
	#2	((TKA OR TKR OR THA OR THR OR total NEXT hip* OR total NEXT knee*) OR ((knee OR knees OR joint OR hip OR hips OR femur* OR femoral) AND (replacement* OR arthroplast* OR artroplast* OR prosthes* OR prosthet* OR alloplast* OR implant*))):ti,ab,kw	24707
	#3	#1 AND #2 in Trials	144
**Cinahl**	S1	(MH "Interns and Residents") OR (MH "Internship and Residency")	29835
	S2	TI ((house N2 officer*) OR resident* OR residency OR trainee* OR registrar* OR (junior N2 surgeon*)) OR AB ((house N2 officer*) OR resident* OR residency OR trainee* OR registrar* OR (junior N2 surgeon*))	110668
	S3	S1 OR S2	122499
	S4	(MH "Arthroplasty, Replacement, Hip") OR (MH "Arthroplasty, Replacement, Knee+")	37056
	S5	TI (TKA OR TKR OR THA OR THR OR total-hip* OR total-knee*) OR AB (TKA OR TKR OR THA OR THR OR total-hip* OR total-knee*)	34558
	S6	S4 OR S5	45639
	S7	(MH "Hip") OR (MH "Knee") OR (MH "Knee Joint+") OR (MH "Hip Joint") OR (MH "Joints+")	92015
	S8	TI (knee OR knees OR joint OR hip OR hips OR femur* OR femoral) OR AB (knee OR knees OR joint OR hip OR hips OR femur* OR femoral)	231570
	S9	S7 OR S8	261457
	S10	(MH "Arthroplasty, Replacement+")	43488
	S11	TI (replacement* OR arthroplast* OR artroplast* OR prosthes* OR prosthet* OR alloplast* OR implant*) OR AB (replacement* OR arthroplast* OR artroplast* OR prosthes* OR prosthet* OR alloplast* OR implant*)	194107
	S12	S10 OR S11	200285
	S13	S9 AND S12	60512
	S14	S6 OR S13	65414
	S15	S3 AND S14	374
**Web of Science**	#1	TOPIC: ((house NEAR/2 officer*) OR resident* OR residency OR trainee* OR registrar* OR (junior NEAR/2 surgeon*))<br>Indexes=SCI-EXPANDED, SSCI, A&HCI, ESCI Timespan=All years	458103
	#2	TOPIC: (TKA OR TKR OR THA OR THR OR total-hip* OR total-knee* OR ((knee OR knees OR joint OR hip OR hips OR femur* OR femoral) AND (replacement* OR arthroplast* OR artroplast* OR prosthes* OR prosthet* OR alloplast* OR implant*)))<br>Indexes=SCI-EXPANDED, SSCI, A&HCI, ESCI Timespan=All years	196746
	#3	#2 AND #1<br>Indexes=SCI-EXPANDED, SSCI, A&HCI, ESCI Timespan=All years	1045

Supplementary Table S2. Reported complication outcomes in methods and results sections in the articles

Study	Reported complication (n for residents/n for consultant) in methods and results section
Lederer 2001	Methods: perioperative Komplikationen (Schaft- und Trochanterfrakturen, Pfannenperforationen, Frühluxationen, klinisch manifeste Nervenläsionen, Thrombosen, Lungenembolien und tiefe Infektionen).<br><br>Results: Fractures (21/24), acetabular perforations (9/10), dislocations (22/34), nerve palsy (26/40), deep infections (27/22), thrombosis, (34/38) pulmonary emboly (14/24)
Moran 2004	Methods: incidence of re-operation, dislocation, deep infection, and death<br><br>Results: Mortality 3 months (2/5), surgical site complications (6/17), deep infection (2/7), fracture (0/1), dislocation (2/7), aseptic loosening (2/1), re-operation (4/11)
Robinson 2007	Methods: registration of “complications”, but type not specified in the methods section<br><br>Results: overall complications (9/7) dislocation (1/2), thrombosis (0/2), pulmonary embolism (0/0), deep infection (0/0), superficial infection (2/2), fracture (0/1), nerve palsy (2/0), hematoma (1/0), **other unrelated to surgery (3/1)**
Woolson 2007	Methods: All intraoperative and postoperative complications were recorded; however, we did not analyze differences in medical complications such as confusion, ileus, urinary complications, and deep venous thrombosis.<br><br>Results:<br>Hip: dislocation (10/7), subluxation (4/1), nerve palsy (1/2), trochanteric fracture (0/2), calcar crack (0/1), hematoma/serious drainage (2/2), early re-operation 0/2), severe heterotopic ossification (3/0), late infection (1/0)<br>Knee: hematoma (2/1), reoperation (0/2), manipulation (1/2), arthrofibrosis (2/0), patellar wear (1/1), insert wear (0/1), cement removal (1/0), extensor re-alignment (0/2), revision (0/1)
Palan 2009	Methods: Secondary outcome measures included complications such as dislocation and revision surgery.<br><br>Results: Dislocation (9/15), revision (7/13)
Inglis 2012	Methods: The revision rate and the reason for revision was summarized. The reason for revision was divided into 6 broad groups: femoral loosening, acetabular loosening, dislocation, infection, femoral fracture, and other.<br><br>Results: revision surgery (396/62) for dislocation (30/147), deep infection (13/60), femoral fracture (4/68), acetabular component loosening (11/44), femoral component loosening (7/41), pain (5/30)
Schoenfeld 2013	Methods: Thirty-day postoperative outcomes included mortality and complications, with complications categorized as major systemic, major local, minor systemic, or minor local. Postoperative infections were also recorded as a separate outcome measure.<br><br>Results: Mortality (13/45) cardiac arrest/myocardial infarction (5/23), pulmonary embolism (45/105), cerebrovascular accident (6/27), acute renal failure (4/15), postoperative sepsis (37/81), septic shock (4/29), **other (33/83)** deep infection (22/71), nerve palsy (8/23), pneumonia (17/79), urinary tract infection (91/289), renal insufficiency (7/29), deep venous thrombosis (65/192), wound dehiscence (16/34), superficial infection (37/148)
Bohl 2014	Methods: mortality, coma >24 hours, on ventilator >48 hours, unplanned intubation, stroke/cerebrovascular accident, pulmonary embolism, cardiac arrest, myocardial infarction, acute renal failure, sepsis, septic shock, or return to the operating room, wound disruption, superficial surgical site infection, deep surgical site infection, organ/space infection (other than surgical site), urinary tract infection, pneumonia, progressive renal insufficiency, graft/prosthesis/flap failure, peripheral nerve injury, and deep vein thrombosis.<br><br>Results: Mortality (8/33), coma >24 hours (1/0), on ventilator >48 hours (9/14), unplanned intubation (15/40), stroke (6/20), pulmonary embolism (38/94), cardiac arrest (4/23), myocardial infarction (15/29), acute renal failure (5/24), sepsis (31/69), septic shock (3/22), return to operating room (63/269), wound disruption (13/32), superficial infection (31/121), deep infection (10/34), organ/space infection (7/23), urinary tract infection (81/256), pneumonia (14/63), renal insufficiency (4/13), **graft/prosthesis/flap failure (5/6),** nerve palsy (7/20), deep venous thrombosis (56/171)
Haughom 2014	Methods: the outcomes identified at thirty days include surgical site infection, wound dehiscence, reoperation, unplanned hospital readmission, pneumonia, unplanned intubation, deep vein thrombosis (DVT), pulmonary embolism (PE), ventilation over 48 hours, renal insufficiency, acute renal failure,

	urinary tract infection, coma, stroke, peripheral neurological deficit, cardiac arrest, myocardial infarction, sepsis, and death.<br><br>Results: Complications (all combined 154/436), surgical complication (58/134) superficial infection (29/73), deep infection (14/28), wound dehiscence (6/10), re-operation (20/37), unplanned re-admission (37/67), **medical complication (103/332),** pneumonia (7/41), unplanned intubation (5/24), deep venous thrombosis (14/49), pulmonary embolism (11/15), ventilated >48h (3/9), renal insufficiency (3/9), acute renal failure (2/9), urinary tract infection (63/147), coma (0/0), stroke (3/17), peripheral neurological deficit (0/0), cardiac arrest (1/8), myocardial infarction (8/18)
Haughom 2014	Methods: outcomes recorded at thirty days postoperatively include surgical site infection, wound dehiscence, pneumonia, unplanned intubation, deep vein thrombosis (DVT), pulmonary embolism (PE), ventilation over 48 hours, renal insufficiency, acute renal failure, urinary tract infection, coma, stroke, peripheral neurological deficit, cardiac arrest, myocardial infarction, sepsis, death, unplanned hospital readmission, and reoperation.<br><br>Results: Complication (all combined 329/949), surgical complication (72/256) superficial infection (41/154), deep infection (8/30), wound dehiscence (15/45), re-operation (17/48), unplanned re-admission (55/163), **medical complication (265/720)**, pneumonia (28/59), unplanned intubation (14/35), deep venous thrombosis (76/199) , pulmonary embolism (49/124), ventilated >48h (9/11), renal insufficiency (5/28), acute renal failure (3/20), urinary tract infection (81/254), coma (1/0), stroke (6/15), peripheral neurological deficit (0/0), cardiac arrest (5/19), myocardial infarction (21/43), sepsis (30/58), death (9/28)
Hasegawa 2015	Methods: registration of "complications", but type not specified in the methods section<br><br>Results: Dislocation (3/1), acetabular fracture (3/1), femoral fracture (/0/1), death (0/0), large vessel injury (2/0), nerve palsy (2/1), deep infection (0/1), skin necrosis (1/0)
Reidy 2016	Methods: complications including dislocation and infection<br><br>Results: re-operations (7/21), deep infections (5/8), dislocations (9/17)
Wilson 2016	Methods: Surgical complications included events such as intraoperative fractures, vessel damage, implant error, broken implant and intraoperative skin tear. Medical complications included events such as respiratory infections, delirium, urinary tract infections and septicaemia. Wound complications included events such as wound ooze, wound infections, wound breakdown and stitch abscess.<br><br>Results:<br>Hip: Surgical complications (51/59), medical complications (113/123), wound complications (113/124), **surgical readmissions 22/26)**<br>Knee: Surgical complications (62/66), medical complications (171/172), wound complications (47/51), **surgical readmissions (47/51)**
Faulkner 2017	Methods: Outcomes including revision rate and mortality were also recorded<br><br>Results: superficial wound infection (0/1), deep wound infection (2/7), superficial infection (0/1), (peri-prosthetic) fracture (0/2), pulmonary embolism (0/5), instability (0/2), malalignment (0/2), limited mobility (0/1), loosening (1/0), reoperation (1/0)
Weber 2017	Methods: registration of "complications", but type not specified in the methods section<br><br>Results: intraoperative femur fracture (1/4), dislocation (0/5), joint infection (2/4)
Weber 2017	Methods: registration of "complications rates", but type not specified in methods section<br><br>Results: intraoperative fractures (0/2), thrombosis (1/1), nerve palsy (1/1), joint infection (1/3)
Windisch 2017	Methods: Early revision reported (for each case the reason for revision was specified (traumatic inlay dislocation, wound healing problems, infection)<br><br>Results: surgical site complications (3/5)
Beattie 2018	Methods: no mentioning of complication in methods section<br><br>Results: Mortality (3/8)
Smith 2018	Methods: revision for early infection<br><br>Results: revision for infection (28/104)
Theelen 2018	Methods: Complication rates were evaluated based on official registration in patient records, with special interest for deep infections

	Results: hypovolemic shock (1/0), hypotension (3/1), cardiac arrhythmia (1/0), pulmonary embolism (0/1), DVT (3/1), superficial surgical site infection (3/7), deep surgical site infection (1/4), wound defect (1/1), erysipelas (1/0), blistering (0/3), patella dislocation (0/2), loosening (1/2), disability in flexion/extension (5/4), peripheral nerve lesion (1/2)
MacDonald 2019	Methods: Patient electronic records were also analyzed to find recorded postoperative complications including 30-day mortality, dislocation, infection requiring reoperation, and revision.<br><br>Results: Mortality (0/0), surgical site complications (0/0), dislocation (0/0), revision (0/0), infection (0/0)
Nakamura 2019	Methods: All adverse events (AEs), serious AEs (such as surgical site infection, cardiovascular events and postoperative fracture), and AEs of special interest (revision surgery, iatrogenic severe sequelae or death) were recorded.<br><br>Results: overall complications (27/15)
Foissey 2020	Methods: major or minor complication (MMC) including intraoperative (e.g., femoral fracture) and post-operative complications (e.g., dislocations, infections, ilio-psoas impingement (IPI), lateral femoral cutaneous nerve (LFCN) neuropraxia)<br><br>Results: Greater trochanteric fractures (3/8), femoral perforation (0/1), periprosthetic fracture (1/4), dislocation (0/1), infection (7/1), aseptic loosening (0/3), psoas pain/iliopsoas impingement (4/18), gluteus tendinitis (8/2), lateral femorocutaneus nerve neuropraxia (10/2), revision (9/10), revision with implant removal (4/4)
Bron 2021	Methods: registration of "complications", but type not specified in the methods section<br><br>Results: Knee: Surgical site (99/222), deep infection (13/45), nerve palsy (5/12), **intra-operative complication (5/10),** re-operation (83/182), **other (69/140),** revision (29/71), systemic complications (91/175), delirium (19/28), **deep venous thrombosis and pulmonary embolism (7/15),** pulmonary (2/11), urological (42/67), cardiac (16/27), gastrointestinal (3/9), stroke (1/10), **other (16/36),** mortality (3/2)<br>Hip: Surgical site (91/248), deep infection (22/50), dislocation (27/68), nerve palsy (8/25), **intra-operative complication (10/29),** re-operation (47/151), **other (26/77),** revision (17/69), systemic complications (116/216), delirium (22/50), **deep venous thrombosis and pulmonary embolism (8/12),** pulmonary (12/23), urological (48/82), cardiac (19/41), gastrointestinal (7/12), stroke (4/6), **other (21/33),** mortality (3/6)
Hoerlesberger 2021	Methods: no mentioning of complication in methods section<br><br>Results: Aseptic loosening (0/2), early infection (1/0)
Sheridan 2022	Methods: all secondary procedures, including revision, re-revision, and the indication for all revision procedures were recorded.<br><br>Results: Mortality (0/0), deep infection (2/2), aseptic loosening (2/2), revision (6/6), manipulation under anesthesia (7/5)
Maheshwari 2022	Methods: Outcomes included 90-day readmission, medical complications including but not limited to deep vein thrombosis, pulmonary embolism, urinary tract infection, cardiopulmonary and gastrointestinal complications, complex regional pain syndrome, and 1-year surgical complications including revision, infections, manipulation under anesthesia, patellar clunk syndrome, and wound issues.<br><br>Results: Deep venous thrombosis (1/0), complex regional pain syndrome (1/1), manipulation under anesthesia (2/2), patella clunk (0/1)
Anis 2022	Methods: Outcomes of interest included surgical site infections and prosthetic joint infections<br><br>Results: Superficial surgical site infection (188/138), prosthetic joint infections (59/25)
Stafford 2023	Methods: Postoperative outcomes were rates of minor complications and infection.<br><br>Results: infections (0/1), other complications (2/5)

All extracted complications from the manuscripts. Complications highlighted in bold were not analyzed separately.

Supplementary Table S3: Forest plots of overall complications*

A: Overall complications*

Study or Subgroup	Residents involved Events	Residents involved Total	No residents involved Events	No residents involved Total	Weight	Risk Ratio M-H, Random, 95% CI	Year
Robinson 2007	9	52	7	83	1.2%	2.05 [0.81, 5.17]	2007
Haughom TKA 2014	154	3462	436	9647	16.6%	0.98 [0.82, 1.18]	2014
Haughom THA 2014	329	5960	949	18569	23.0%	1.08 [0.96, 1.22]	2014
Wilson 2016	609	2313	679	2605	26.5%	1.01 [0.92, 1.11]	2016
Faulkner 2017	1	236	22	450	0.3%	0.09 [0.01, 0.64]	2017
Theelen 2018	21	220	33	422	3.4%	1.22 [0.72, 2.06]	2018
Nakamura 2019	27	58	15	42	3.8%	1.30 [0.80, 2.13]	2019
Bron 2021	397	2027	861	5096	24.8%	1.16 [1.04, 1.29]	2020
Stafford 2023	2	100	5	100	0.4%	0.40 [0.08, 2.01]	2023
**Total (95% CI)**		**14428**		**37014**	**100.0%**	**1.07 [0.97, 1.19]**	
Total events	1549		3007				

Heterogeneity: Tau² = 0.01; Chi² = 14.76, df = 8 (P = 0.06); I² = 46%
Test for overall effect: Z = 1.35 (P = 0.18)

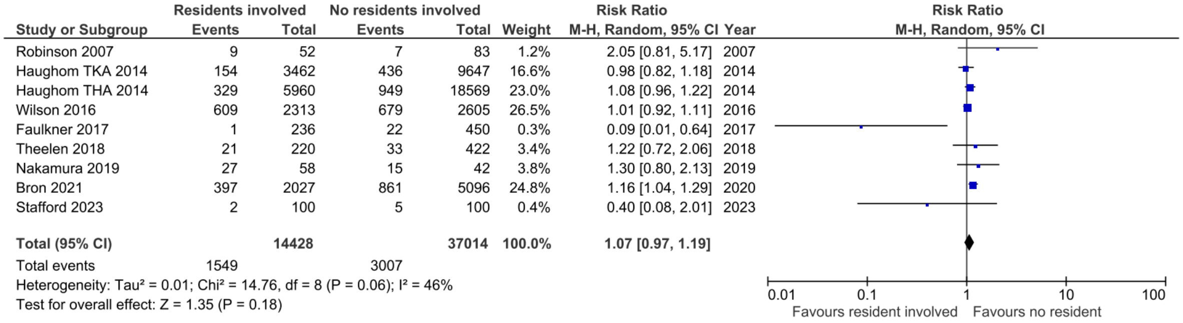

B: Surgical complications*

Study or Subgroup	Residents involved Events	Residents involved Total	No residents involved Events	No residents involved Total	Weight	Risk Ratio M-H, Random, 95% CI	Year
Moran 2004	6	139	17	397	3.1%	1.01 [0.41, 2.51]	2004
Woolson 2007	29	191	39	210	10.2%	0.82 [0.53, 1.27]	2007
Bohl 2014	129	4260	482	17174	23.0%	1.08 [0.89, 1.31]	2014
Wilson 2016	113	2313	125	2605	19.1%	1.02 [0.79, 1.30]	2016
Faulkner 2017	1	236	16	450	0.7%	0.12 [0.02, 0.89]	2017
Windisch 2017	3	80	5	988	1.4%	7.41 [1.80, 30.45]	2017
Theelen 2018	12	220	25	422	5.3%	0.92 [0.47, 1.80]	2018
MacDonald 2019	0	44	0	43		Not estimable	2019
Bron 2021	190	2027	470	5096	25.2%	1.02 [0.87, 1.19]	2020
Foissey 2020	30	147	50	341	11.2%	1.39 [0.92, 2.10]	2020
Hoerlesberger 2021	1	103	2	103	0.5%	0.50 [0.05, 5.43]	2021
Stafford 2023	0	100	1	100	0.3%	0.33 [0.01, 8.09]	2023
**Total (95% CI)**		**9860**		**27929**	**100.0%**	**1.04 [0.88, 1.24]**	
Total events	514		1232				

Heterogeneity: Tau² = 0.02; Chi² = 16.27, df = 10 (P = 0.09); I² = 39%
Test for overall effect: Z = 0.51 (P = 0.61)

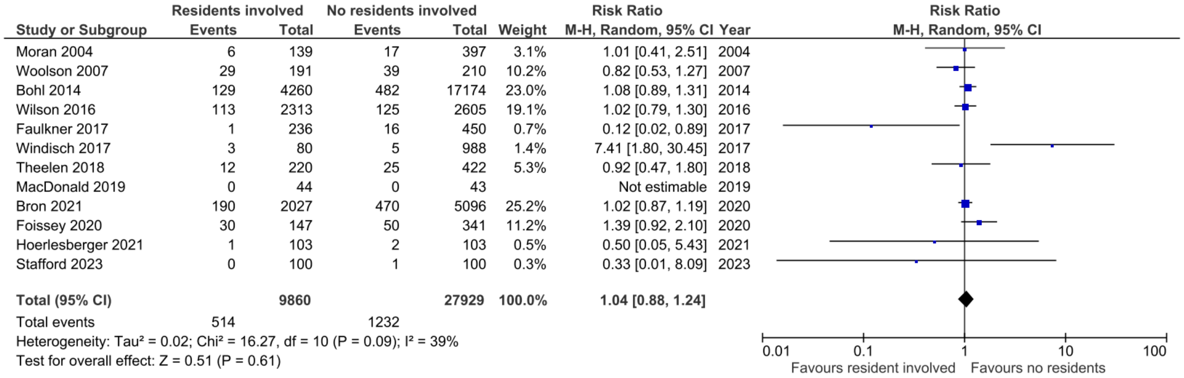

C: Mortality*

Study or Subgroup	Residents involved Events	Residents involved Total	No residents involved Events	No residents involved Total	Weight	Risk Ratio M-H, Random, 95% CI	Year
Moran 2004	2	139	5	397	8.4%	1.14 [0.22, 5.82]	2004
Schoenfeld 2013	13	4816	45	18967	58.7%	1.14 [0.61, 2.11]	2013
Beattie 2018	3	144	8	465	12.9%	1.21 [0.33, 4.50]	2018
MacDonald 2019	0	44	0	43		Not estimable	2019
Bron 2021	6	2027	8	5096	20.0%	1.89 [0.66, 5.43]	2020
Sheridan 2022	0	315	0	315		Not estimable	2022
**Total (95% CI)**		**7485**		**25283**	**100.0%**	**1.27 [0.79, 2.04]**	
Total events	24		66				

Heterogeneity: Tau² = 0.00; Chi² = 0.68, df = 3 (P = 0.88); I² = 0%
Test for overall effect: Z = 0.99 (P = 0.32)

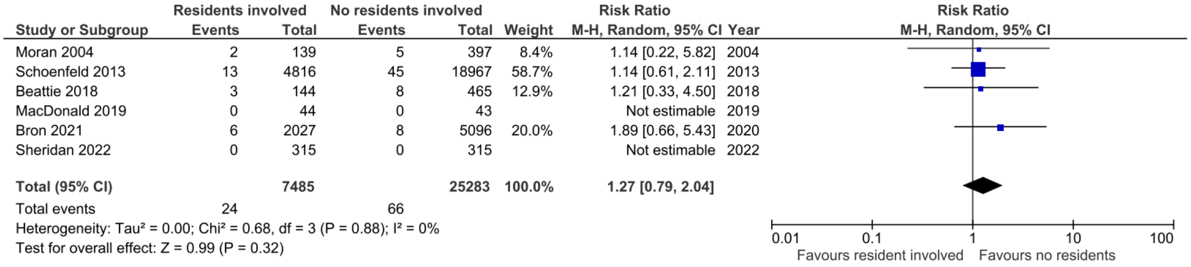

* The 4 NSQIP studies (13-16) had overlapping cohorts. For A, both studies of Haughom et al. were included since they also included the cohorts of Bohl et al. and Schoenfeld et al. For B, the study by Bohl et al. was included since it reported the highest number of surgical complications from all NSQIP studies. For C, the study by Schoenfeld et al. was included since it reported the highest number of deaths from all NSQIP studies.

Supplementary Table S4: Forest plots of surgical site complications

A: Deep infection*

Study or Subgroup	Residents involved Events	Residents involved Total	No residents involved Events	No residents involved Total	Weight	Risk Ratio M-H, Random, 95% CI	Year
Lederer 2001	27	1290	11	2000	11.0%	3.81 [1.89, 7.64]	2001
Moran 2004	2	139	7	397	4.0%	0.82 [0.17, 3.88]	2004
Robinson 2007	0	52	0	83		Not estimable	2007
Woolson 2007	1	119	0	210	1.1%	5.28 [0.22, 128.47]	2007
Schoenfeld 2013	22	4816	71	18967	14.2%	1.22 [0.76, 1.97]	2013
Hasegawa 2015	0	259	1	224	1.1%	0.29 [0.01, 7.05]	2015
Reidy 2016	5	286	8	584	6.6%	1.28 [0.42, 3.87]	2016
Windisch 2017	0	80	1	988	1.1%	4.07 [0.17, 99.11]	2017
Weber TKA 2017	1	292	3	446	2.1%	0.51 [0.05, 4.87]	2017
Faulkner 2017	2	236	7	450	4.0%	0.54 [0.11, 2.60]	2017
Weber THA 2017	2	240	4	768	3.5%	1.60 [0.29, 8.68]	2017
Theelen 2018	1	220	4	422	2.3%	0.48 [0.05, 4.26]	2018
Smith 2018	28	9586	104	79501	15.2%	2.23 [1.47, 3.39]	2018
MacDonald 2019	0	44	0	43		Not estimable	2019
Foissey 2020	7	147	1	341	2.5%	16.24 [2.02, 130.80]	2020
Bron 2021	35	2027	95	5096	15.7%	0.93 [0.63, 1.36]	2020
Hoerlesberger 2021	1	103	0	103	1.1%	3.00 [0.12, 72.80]	2021
Anis 2022	59	7234	25	5430	14.4%	1.77 [1.11, 2.82]	2022
**Total (95% CI)**		**27170**		**116053**	**100.0%**	**1.55 [1.09, 2.20]**	
Total events	193		342				

Heterogeneity: Tau² = 0.17; Chi² = 28.99, df = 15 (P = 0.02); I² = 48%
Test for overall effect: Z = 2.44 (P = 0.01)

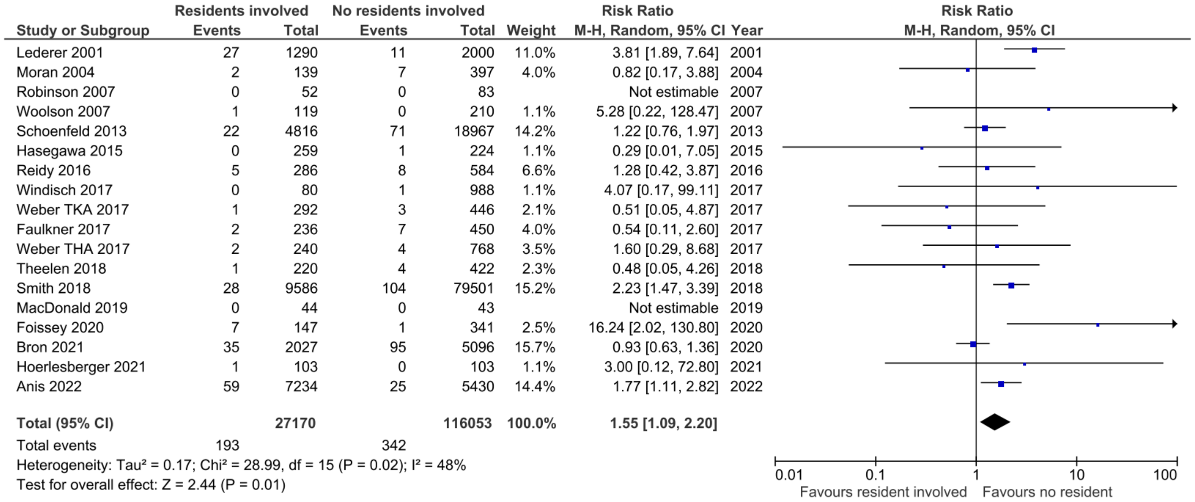

B: Re-operation*

Study or Subgroup	Residents involved Events	Residents involved Total	No residents involved Events	No residents involved Total	Weight	Risk Ratio M-H, Random, 95% CI	Year
Moran 2004	4	139	11	397	2.6%	1.04 [0.34, 3.21]	2004
Woolson 2007	2	191	6	210	1.3%	0.37 [0.07, 1.79]	2007
Palan 2009	7	528	13	973	3.9%	0.99 [0.40, 2.47]	2009
Inglis 2012	62	4049	396	30344	25.6%	1.17 [0.90, 1.53]	2012
Haughom THA 2014	20	3462	37	9647	9.7%	1.51 [0.88, 2.59]	2014
Haughom TKA 2014	17	5960	48	18569	9.4%	1.10 [0.64, 1.92]	2014
Reidy 2016	7	286	21	584	4.5%	0.68 [0.29, 1.58]	2016
Windisch 2017	2	80	3	988	1.1%	8.23 [1.40, 48.56]	2017
Faulkner 2017	1	236	0	450	0.3%	5.71 [0.23, 139.60]	2017
Theelen 2018	2	220	6	422	1.3%	0.64 [0.13, 3.14]	2018
MacDonald 2019	0	44	0	43		Not estimable	2019
Foissey 2020	9	147	10	341	4.1%	2.09 [0.87, 5.03]	2020
Bron 2021	130	2027	333	5096	33.5%	0.98 [0.81, 1.19]	2020
Sheridan 2022	6	315	6	315	2.6%	1.00 [0.33, 3.07]	2022
**Total (95% CI)**		**17684**		**68379**	**100.0%**	**1.11 [0.92, 1.34]**	
Total events	269		890				

Heterogeneity: Tau² = 0.02; Chi² = 14.39, df = 12 (P = 0.28); I² = 17%
Test for overall effect: Z = 1.12 (P = 0.26)

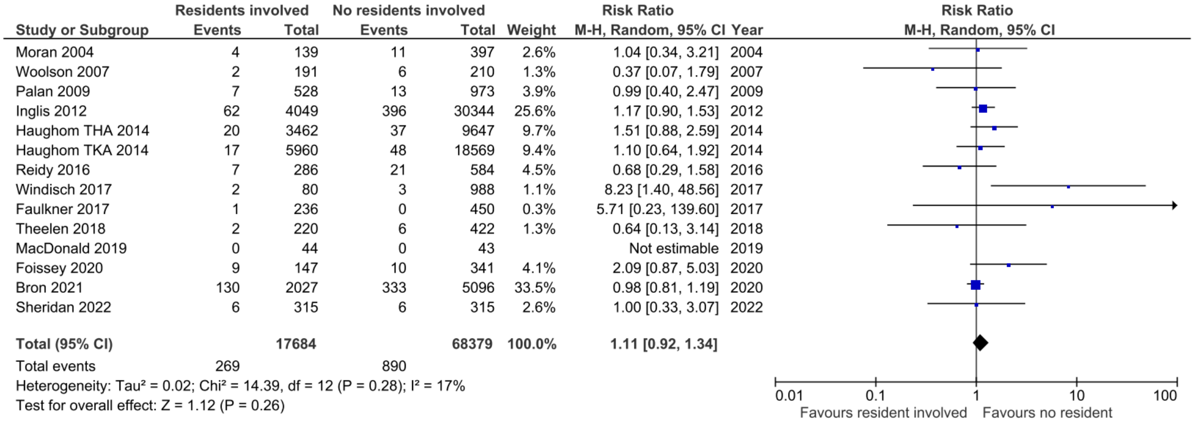

C: Dislocation total hip arthroplasty

Study or Subgroup	Residents involved Events	Residents involved Total	No residents involved Events	No residents involved Total	Weight	Risk Ratio M-H, Random, 95% CI	Year
Lederer 2001	22	1290	34	2000	17.3%	1.00 [0.59, 1.71]	2001
Moran 2004	2	139	7	397	2.0%	0.82 [0.17, 3.88]	2004
Robinson 2007	1	52	2	83	0.9%	0.80 [0.07, 8.58]	2007
Woolson 2007	10	119	7	111	5.6%	1.33 [0.53, 3.38]	2007
Palan 2009	9	528	15	973	7.3%	1.11 [0.49, 2.51]	2009
Inglis 2012	30	4049	147	30344	31.9%	1.53 [1.03, 2.26]	2012
Hasegawa 2015	3	259	1	224	1.0%	2.59 [0.27, 24.77]	2015
Reidy 2016	9	286	17	584	7.7%	1.08 [0.49, 2.40]	2016
Weber THA 2017	0	240	5	768	0.6%	0.29 [0.02, 5.23]	2017
MacDonald 2019	0	44	0	43		Not estimable	2019
Foissey 2020	0	147	1	341	0.5%	0.77 [0.03, 18.80]	2020
Bron 2021	27	1151	68	2878	25.2%	0.99 [0.64, 1.54]	2020
**Total (95% CI)**		**8304**		**38746**	**100.0%**	**1.17 [0.94, 1.46]**	
Total events	113		304				

Heterogeneity: Tau² = 0.00; Chi² = 4.55, df = 10 (P = 0.92); I² = 0%
Test for overall effect: Z = 1.41 (P = 0.16)

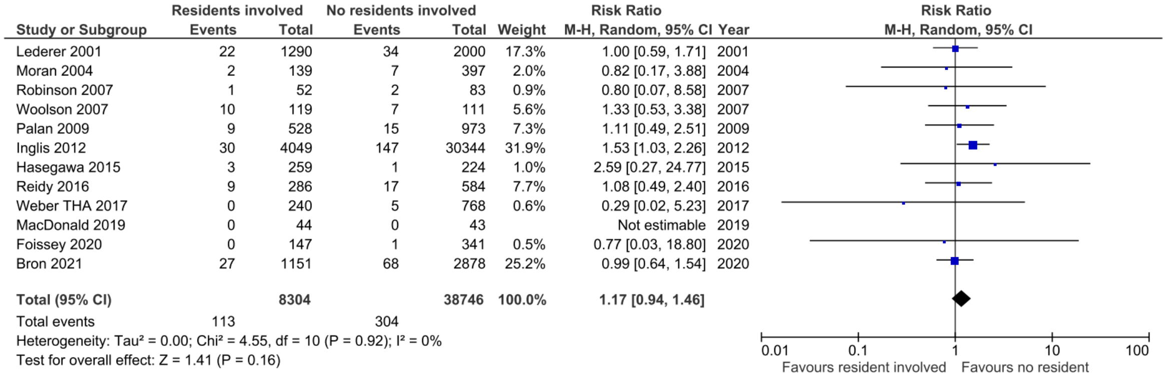

D: Fracture

Study or Subgroup	Residents involved Events	Residents involved Total	No residents involved Events	No residents involved Total	Weight	Risk Ratio M-H, Random, 95% CI	Year
Lederer 2001	21	1290	24	2000	51.7%	1.36 [0.76, 2.43]	2001
Moran 2004	0	139	1	397	1.7%	0.95 [0.04, 23.13]	2004
Woolson 2007	0	191	3	210	2.0%	0.16 [0.01, 3.02]	2007
Inglis 2012	4	4049	68	30344	17.2%	0.44 [0.16, 1.21]	2012
Hasegawa 2015	3	259	2	224	5.5%	1.30 [0.22, 7.69]	2015
Weber THA 2017	1	240	4	768	3.7%	0.80 [0.09, 7.12]	2017
Weber TKA 2017	0	292	2	446	1.9%	0.31 [0.01, 6.33]	2017
Faulkner 2017	0	236	2	450	1.9%	0.38 [0.02, 7.90]	2017
Foissey 2020	4	147	13	341	14.4%	0.71 [0.24, 2.15]	2020
**Total (95% CI)**		**6843**		**35180**	**100.0%**	**0.90 [0.59, 1.37]**	
Total events	33		119				

Heterogeneity: Tau² = 0.00; Chi² = 6.49, df = 8 (P = 0.59); I² = 0%
Test for overall effect: Z = 0.49 (P = 0.62)

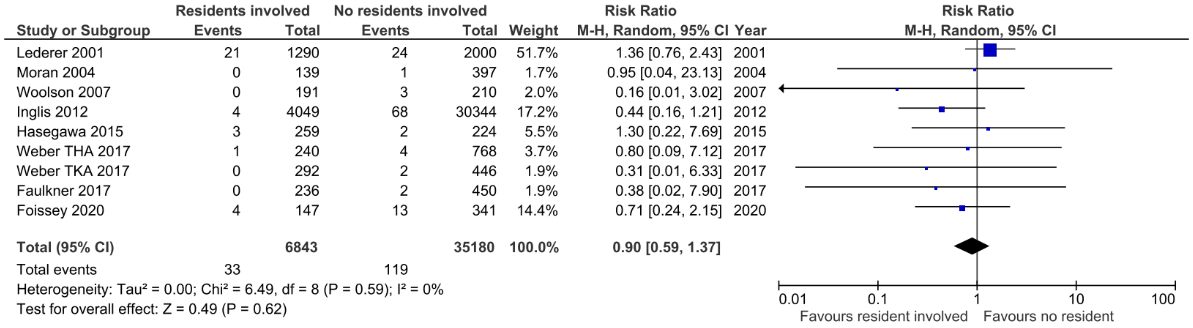

E: Nerve palsy*

Study or Subgroup	Residents involved Events	Residents involved Total	No residents involved Events	No residents involved Total	Weight	Risk Ratio M-H, Random, 95% CI	Year
Lederer 2001	26	1290	40	2000	28.0%	1.01 [0.62, 1.64]	2001
Woolson 2007	1	119	2	111	4.5%	0.47 [0.04, 5.07]	2007
Robinson 2007	2	52	0	83	2.9%	7.92 [0.39, 161.87]	2007
Schoenfeld 2013	8	4816	23	18967	20.1%	1.37 [0.61, 3.06]	2013
Hasegawa 2015	2	259	0	224	2.9%	4.33 [0.21, 89.66]	2015
Weber TKA 2017	1	292	1	446	3.4%	1.53 [0.10, 24.32]	2017
Theelen 2018	1	220	2	422	4.4%	0.96 [0.09, 10.52]	2018
Foissey 2020	10	147	2	341	9.5%	11.60 [2.57, 52.28]	2020
Bron 2021	13	2027	37	5096	24.3%	0.88 [0.47, 1.66]	2020
**Total (95% CI)**		**9222**		**27690**	**100.0%**	**1.42 [0.83, 2.43]**	
Total events	64		107				

Heterogeneity: Tau² = 0.21; Chi² = 13.21, df = 8 (P = 0.10); I² = 39%
Test for overall effect: Z = 1.27 (P = 0.20)

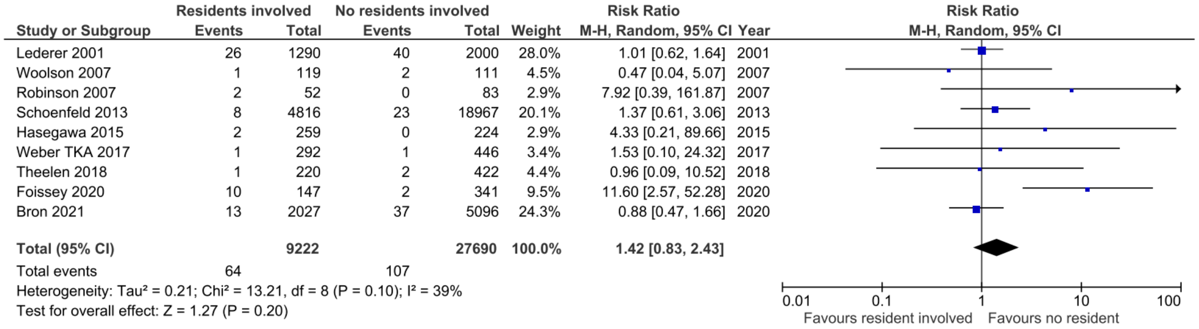

F: Superficial infection*

Study or Subgroup	Residents involved Events	Residents involved Total	No residents involved Events	No residents involved Total	Weight	Risk Ratio M-H, Random, 95% CI	Year
Robinson 2007	2	52	2	83	0.7%	1.60 [0.23, 10.99]	2007
Haughom TKA 2014	29	3462	73	9647	15.0%	1.11 [0.72, 1.70]	2014
Haughom THA 2014	41	5960	154	18569	23.4%	0.83 [0.59, 1.17]	2014
Windisch 2017	1	80	1	988	0.4%	12.35 [0.78, 195.60]	2017
Faulkner 2017	0	236	1	450	0.3%	0.63 [0.03, 15.51]	2017
Theelen 2018	3	220	7	422	1.5%	0.82 [0.21, 3.15]	2018
Anis 2022	188	7234	138	5430	58.7%	1.02 [0.82, 1.27]	2022
**Total (95% CI)**		**17244**		**35589**	**100.0%**	**0.99 [0.84, 1.17]**	
Total events	264		376				

Heterogeneity: Tau² = 0.00; Chi² = 4.96, df = 6 (P = 0.55); I² = 0%
Test for overall effect: Z = 0.08 (P = 0.93)

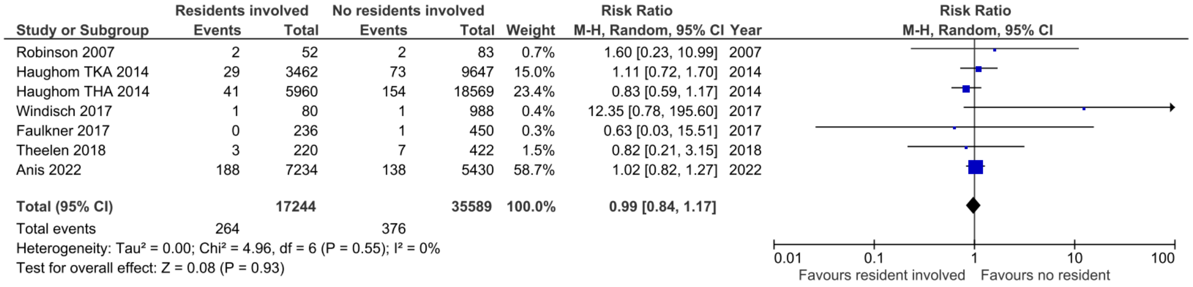

G: Aseptic loosening

Study or Subgroup	Residents involved Events	Residents involved Total	No residents involved Events	No residents involved Total	Weight	Risk Ratio M-H, Random, 95% CI	Year
Moran 2004	2	139	1	397	3.6%	5.71 [0.52, 62.51]	2004
Inglis 2012	18	4049	85	30344	80.6%	1.59 [0.96, 2.64]	2012
Faulkner 2017	1	236	0	450	2.0%	5.71 [0.23, 139.60]	2017
Theelen 2018	1	220	2	422	3.6%	0.96 [0.09, 10.52]	2018
Foissey 2020	0	147	3	341	2.4%	0.33 [0.02, 6.35]	2020
Hoerlesberger 2021	0	103	2	103	2.3%	0.20 [0.01, 4.12]	2021
Sheridan 2022	2	315	2	315	5.4%	1.00 [0.14, 7.05]	2022
**Total (95% CI)**		**5209**		**32372**	**100.0%**	**1.50 [0.95, 2.37]**	
Total events	24		95				

Heterogeneity: Tau² = 0.00; Chi² = 4.98, df = 6 (P = 0.55); I² = 0%
Test for overall effect: Z = 1.75 (P = 0.08)

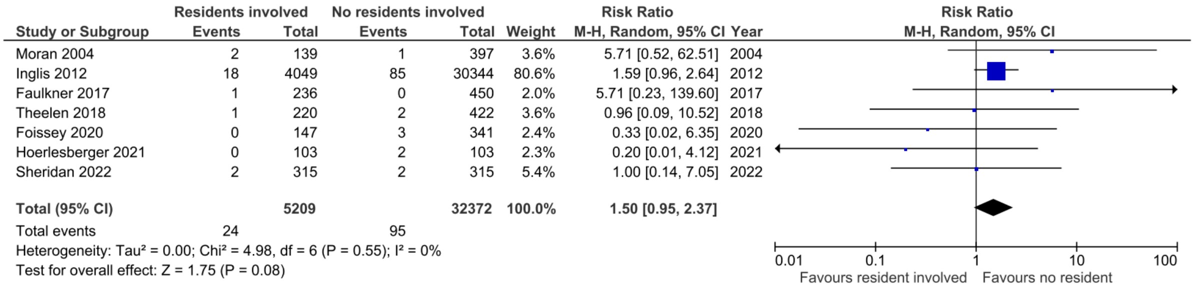

H: Limited mobility total knee arthroplasty

Study or Subgroup	Residents involved Events	Residents involved Total	No residents involved Events	No residents involved Total	Weight	Risk Ratio M-H, Random, 95% CI	Year
Woolson 2007	3	72	2	99	15.7%	2.06 [0.35, 12.03]	2007
Faulkner 2017	0	236	1	450	4.8%	0.63 [0.03, 15.51]	2017
Theelen 2018	5	220	4	422	28.6%	2.40 [0.65, 8.84]	2018
Sheridan 2022	7	315	5	315	37.7%	1.40 [0.45, 4.36]	2022
Maheshwari 2022	2	50	2	50	13.2%	1.00 [0.15, 6.82]	2022
**Total (95% CI)**		**893**		**1336**	**100.0%**	**1.60 [0.80, 3.21]**	
Total events	17		14				

Heterogeneity: Tau² = 0.00; Chi² = 1.05, df = 4 (P = 0.90); I² = 0%
Test for overall effect: Z = 1.32 (P = 0.19)

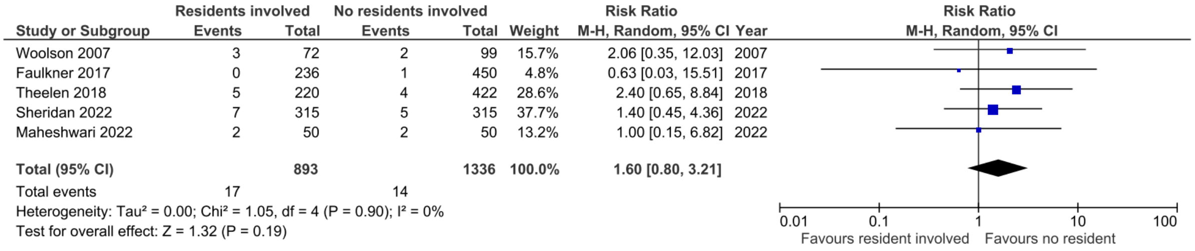

I: Instability total knee arthroplasty

Study or Subgroup	Residents involved Events	Residents involved Total	No residents involved Events	No residents involved Total	Weight	Risk Ratio M-H, Random, 95% CI	Year
Windisch 2017	0	236	2	450	43.2%	0.38 [0.02, 7.90]	2017
Faulkner 2017	2	80	3	988	56.8%	8.23 [1.40, 48.56]	2017
**Total (95% CI)**		**316**		**1438**	**100.0%**	**2.18 [0.08, 58.95]**	
Total events	2		5				

Heterogeneity: Tau² = 4.16; Chi² = 3.59, df = 1 (P = 0.06); I² = 72%
Test for overall effect: Z = 0.46 (P = 0.64)

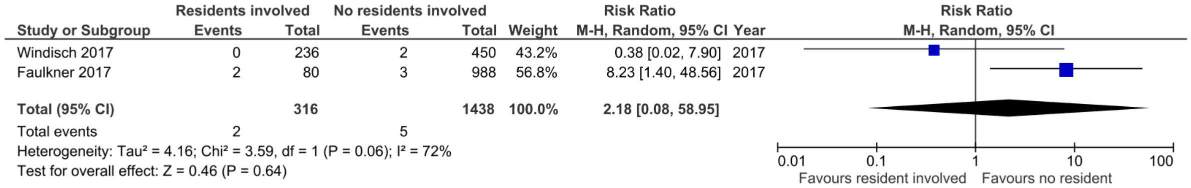

J: Wound defect*

Study or Subgroup	Residents involved Events	Residents involved Total	No residents involved Events	No residents involved Total	Weight	Risk Ratio M-H, Random, 95% CI	Year
Haughom TKA 2014	15	5960	45	18569	70.9%	1.04 [0.58, 1.86]	2014
Haughom THA 2014	6	3462	10	9647	23.6%	1.67 [0.61, 4.60]	2014
Hasegawa 2015	1	259	0	224	2.4%	2.60 [0.11, 63.41]	2015
Theelen 2018	1	220	1	422	3.2%	1.92 [0.12, 30.52]	2018
**Total (95% CI)**		**9901**		**28862**	**100.0%**	**1.21 [0.74, 1.98]**	
Total events	23		56				

Heterogeneity: Tau² = 0.00; Chi² = 0.98, df = 3 (P = 0.81); I² = 0%
Test for overall effect: Z = 0.76 (P = 0.45)

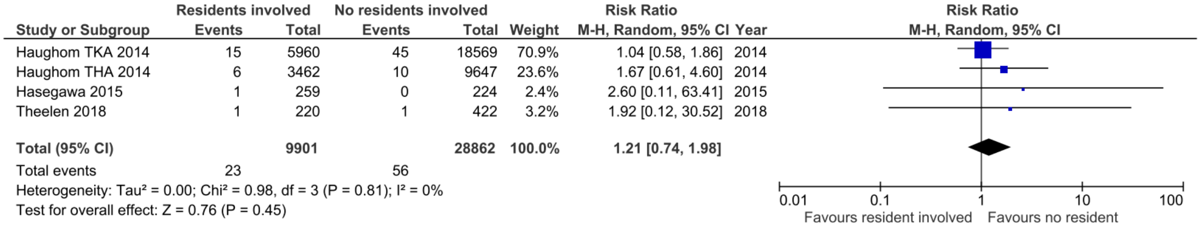

K: Malalignment total knee arthroplasty

Study or Subgroup	Residents involved Events	Residents involved Total	No residents involved Events	No residents involved Total	Weight	Risk Ratio M-H, Random, 95% CI	Year
Woolson 2007	0	72	2	99	50.2%	0.27 [0.01, 5.62]	2007
Faulkner 2017	0	236	2	450	49.8%	0.38 [0.02, 7.90]	2017
**Total (95% CI)**		**308**		**549**	**100.0%**	**0.32 [0.04, 2.74]**	
Total events	0		4				

Heterogeneity: Tau² = 0.00; Chi² = 0.02, df = 1 (P = 0.88); I² = 0%
Test for overall effect: Z = 1.04 (P = 0.30)

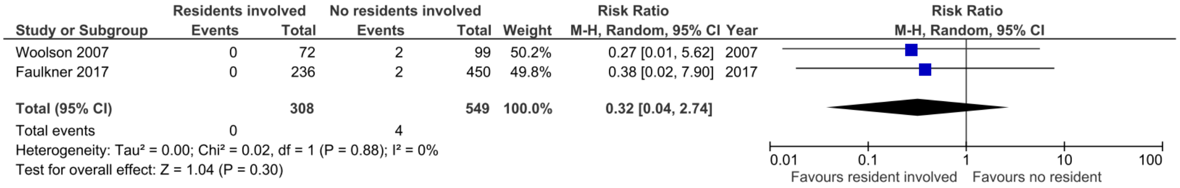

L: Serious drainage / hematoma

Study or Subgroup	Residents involved Events	Residents involved Total	No residents involved Events	No residents involved Total	Weight	Risk Ratio M-H, Random, 95% CI	Year
Robinson 2007	1	52	0	83	17.9%	4.75 [0.20, 114.57]	2007
Woolson 2007	4	191	3	210	82.1%	1.47 [0.33, 6.47]	2007
**Total (95% CI)**		**243**		**293**	**100.0%**	**1.81 [0.47, 6.94]**	
Total events	5		3				

Heterogeneity: Tau² = 0.00; Chi² = 0.43, df = 1 (P = 0.51); I² = 0%
Test for overall effect: Z = 0.86 (P = 0.39)

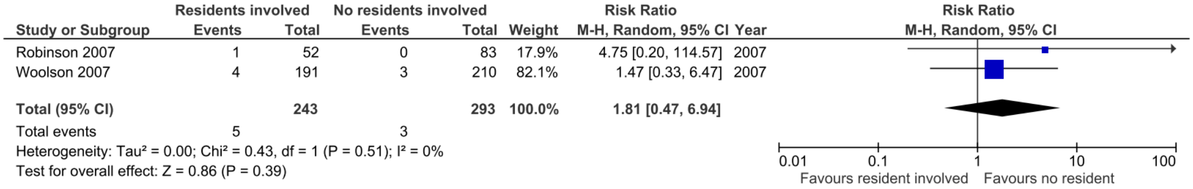

* The 4 NSQIP studies (13-16) had overlapping cohorts. For A and L, both studies of Haughom et al. were included since they also included the cohorts of Bohl et al. and Schoenfeld et al. For B and J, the study by Schoenfeld et al. was included since it reported the highest number of deep infections and nerve palsies from all NSQIP studies.

Supplementary Table S5: Forest plots of systemic complications

A: On ventilator >48 hours*

Study or Subgroup	Residents involved Events	Residents involved Total	No residents involved Events	No residents involved Total	Weight	Risk Ratio M-H, Fixed, 95% CI
Haughom THA 2014	5	3462	24	9647	42.7%	0.58 [0.22, 1.52]
Haughom TKA 2014	14	5960	35	18569	57.3%	1.25 [0.67, 2.31]
**Total (95% CI)**		**9422**		**28216**	**100.0%**	**0.96 [0.57, 1.61]**
Total events	19		59			

Heterogeneity: Chi² = 1.73, df = 1 (P = 0.19); I² = 42%
Test for overall effect: Z = 0.15 (P = 0.88)

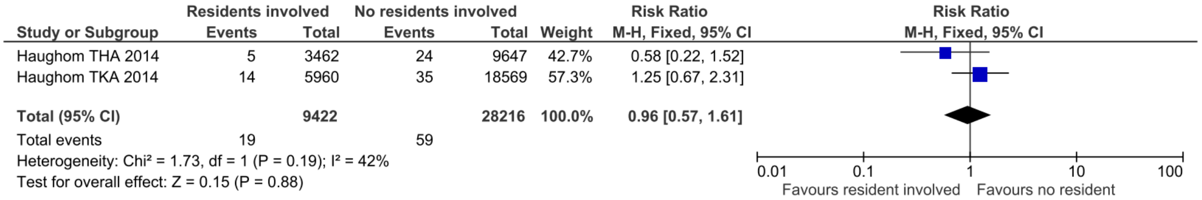

B: Unplanned intubation*

Study or Subgroup	Residents involved Events	Residents involved Total	No residents involved Events	No residents involved Total	Weight	Risk Ratio M-H, Fixed, 95% CI
Haughom THA 2014	5	3462	24	9647	42.7%	0.58 [0.22, 1.52]
Haughom TKA 2014	14	5960	35	18569	57.3%	1.25 [0.67, 2.31]
**Total (95% CI)**		**9422**		**28216**	**100.0%**	**0.96 [0.57, 1.61]**
Total events	19		59			

Heterogeneity: Chi² = 1.73, df = 1 (P = 0.19); I² = 42%
Test for overall effect: Z = 0.15 (P = 0.88)

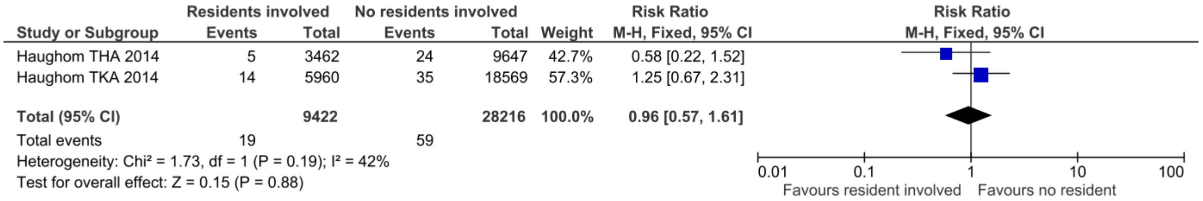

C: Pneumonia*

Study or Subgroup	Residents involved Events	Residents involved Total	No residents involved Events	No residents involved Total	Weight	Risk Ratio M-H, Random, 95% CI	Year
Haughom TKA 2014	28	5960	59	18569	54.3%	1.48 [0.94, 2.32]	2014
Haughom THA 2014	7	3462	41	9647	45.7%	0.48 [0.21, 1.06]	2014
**Total (95% CI)**		**9422**		**28216**	**100.0%**	**0.88 [0.29, 2.70]**	
Total events	35		100				

Heterogeneity: Tau² = 0.55; Chi² = 6.00, df = 1 (P = 0.01); I² = 83%
Test for overall effect: Z = 0.22 (P = 0.82)

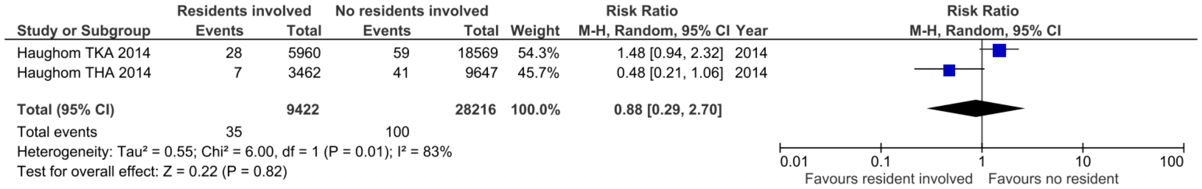

D: Cardiac arrest*

Study or Subgroup	Residents involved Events	Residents involved Total	No residents involved Events	No residents involved Total	Weight	Risk Ratio M-H, Random, 95% CI	Year
Haughom TKA 2014	5	5960	19	18569	81.7%	0.82 [0.31, 2.19]	2014
Haughom THA 2014	1	3462	8	9647	18.3%	0.35 [0.04, 2.78]	2014
**Total (95% CI)**		**9422**		**28216**	**100.0%**	**0.70 [0.29, 1.71]**	
Total events	6		27				

Heterogeneity: Tau² = 0.00; Chi² = 0.54, df = 1 (P = 0.46); I² = 0%
Test for overall effect: Z = 0.78 (P = 0.43)

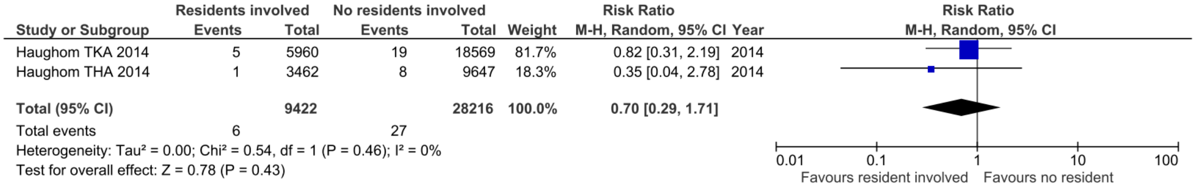

E: Myocardial infarction*

Study or Subgroup	Residents involved Events	Residents involved Total	No residents involved Events	No residents involved Total	Weight	Risk Ratio M-H, Random, 95% CI	Year
Haughom TKA 2014	21	5960	43	18569	71.8%	1.52 [0.90, 2.56]	2014
Haughom THA 2014	8	3462	18	9647	28.2%	1.24 [0.54, 2.85]	2014
**Total (95% CI)**		**9422**		**28216**	**100.0%**	**1.44 [0.92, 2.23]**	
Total events	29		61				

Heterogeneity: Tau² = 0.00; Chi² = 0.17, df = 1 (P = 0.68); I² = 0%
Test for overall effect: Z = 1.61 (P = 0.11)

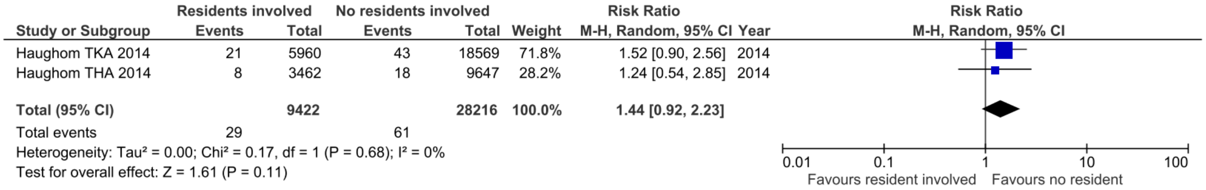

F: Deep venous thrombosis*

Study or Subgroup	Residents involved Events	Residents involved Total	No residents involved Events	No residents involved Total	Weight	Risk Ratio M-H, Random, 95% CI	Year
Lederer 2001	34	1290	38	2000	21.3%	1.39 [0.88, 2.19]	2001
Robinson 2007	0	52	2	83	0.5%	0.32 [0.02, 6.47]	2007
Haughom THA 2014	14	3462	49	9647	12.7%	0.80 [0.44, 1.44]	2014
Haughom TKA 2014	76	5960	199	18569	64.5%	1.19 [0.92, 1.55]	2014
Weber TKA 2017	1	292	1	446	0.6%	1.53 [0.10, 24.32]	2017
Maheshwari 2022	1	50	0	50	0.4%	3.00 [0.13, 71.92]	2022
**Total (95% CI)**		**11106**		**30795**	**100.0%**	**1.17 [0.95, 1.44]**	
Total events	126		289				

Heterogeneity: Tau² = 0.00; Chi² = 3.27, df = 5 (P = 0.66); I² = 0%
Test for overall effect: Z = 1.44 (P = 0.15)

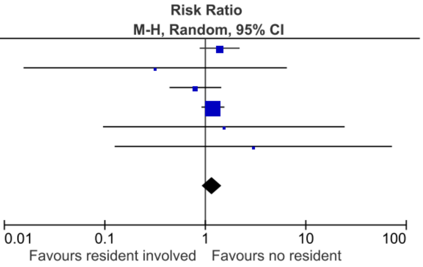

G: Pulmonary embolism*

Study or Subgroup	Residents involved Events	Residents involved Total	No residents involved Events	No residents involved Total	Weight	Risk Ratio M-H, Random, 95% CI	Year
Lederer 2001	14	1290	24	2000	21.4%	0.90 [0.47, 1.74]	2001
Robinson 2007	0	52	0	83		Not estimable	2007
Haughom TKA 2014	49	5960	124	18569	60.4%	1.23 [0.89, 1.71]	2014
Haughom THA 2014	11	3462	15	9647	15.9%	2.04 [0.94, 4.44]	2014
Faulkner 2017	0	236	5	450	1.3%	0.17 [0.01, 3.12]	2017
Theelen 2018	0	220	1	422	1.0%	0.64 [0.03, 15.60]	2018
**Total (95% CI)**		**11220**		**31171**	**100.0%**	**1.21 [0.87, 1.68]**	
Total events	74		169				

Heterogeneity: Tau² = 0.02; Chi² = 4.44, df = 4 (P = 0.35); I² = 10%
Test for overall effect: Z = 1.14 (P = 0.25)

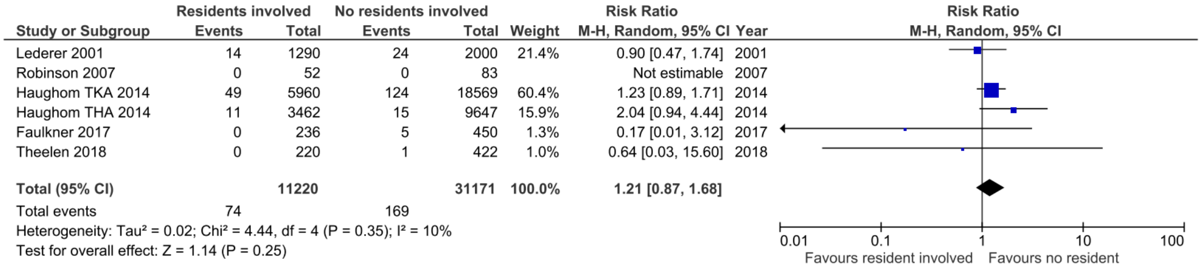

H: Cerebrovascular accident*

Study or Subgroup	Residents involved Events	Residents involved Total	No residents involved Events	No residents involved Total	Weight	Risk Ratio M-H, Random, 95% CI	Year
Haughom THA 2014	3	3462	17	9647	23.9%	0.49 [0.14, 1.68]	2014
Haughom TKA 2014	6	5960	15	18569	40.2%	1.25 [0.48, 3.21]	2014
Bron 2021	5	2027	16	5096	35.8%	0.79 [0.29, 2.14]	2020
**Total (95% CI)**		**11449**		**33312**	**100.0%**	**0.85 [0.46, 1.54]**	
Total events	14		48				

Heterogeneity: Tau² = 0.00; Chi² = 1.43, df = 2 (P = 0.49); I² = 0%
Test for overall effect: Z = 0.55 (P = 0.58)

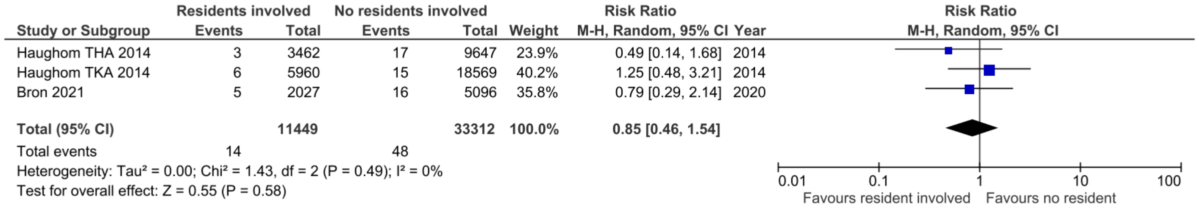

I: Renal insufficiency*

Study or Subgroup	Residents involved Events	Residents involved Total	No residents involved Events	No residents involved Total	Weight	Risk Ratio M-H, Fixed, 95% CI
Haughom THA 2014	3	3462	9	9647	25.9%	0.93 [0.25, 3.43]
Haughom TKA 2014	5	5960	28	18569	74.1%	0.56 [0.21, 1.44]
**Total (95% CI)**		**9422**		**28216**	**100.0%**	**0.65 [0.30, 1.40]**
Total events	8		37			

Heterogeneity: Chi² = 0.39, df = 1 (P = 0.53); I² = 0%
Test for overall effect: Z = 1.09 (P = 0.27)

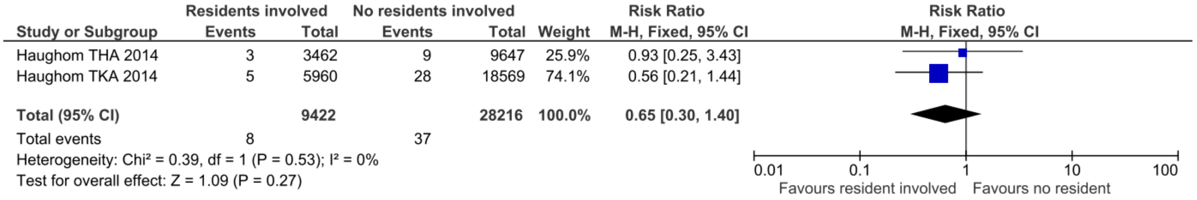

J: Acute renal failure*

Study or Subgroup	Residents involved Events	Residents involved Total	No residents involved Events	No residents involved Total	Weight	Risk Ratio M-H, Random, 95% CI	Year
Haughom TKA 2014	3	5960	20	18569	61.5%	0.47 [0.14, 1.57]	2014
Haughom THA 2014	2	3462	9	9647	38.5%	0.62 [0.13, 2.86]	2014
**Total (95% CI)**		**9422**		**28216**	**100.0%**	**0.52 [0.20, 1.35]**	
Total events	5		29				

Heterogeneity: Tau² = 0.00; Chi² = 0.08, df = 1 (P = 0.78); I² = 0%
Test for overall effect: Z = 1.34 (P = 0.18)

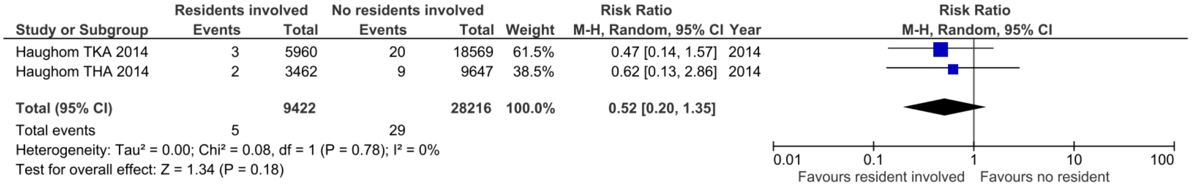

K: Urinary tract infection*

Study or Subgroup	Residents involved Events	Residents involved Total	No residents involved Events	No residents involved Total	Weight	Risk Ratio M-H, Random, 95% CI	Year
Haughom TKA 2014	81	5960	254	18569	58.1%	0.99 [0.78, 1.27]	2014
Haughom THA 2014	63	3462	147	9647	41.9%	1.19 [0.89, 1.60]	2014
**Total (95% CI)**		**9422**		**28216**	**100.0%**	**1.07 [0.89, 1.30]**	
Total events	144		401				

Heterogeneity: Tau² = 0.00; Chi² = 0.88, df = 1 (P = 0.35); I² = 0%
Test for overall effect: Z = 0.73 (P = 0.46)

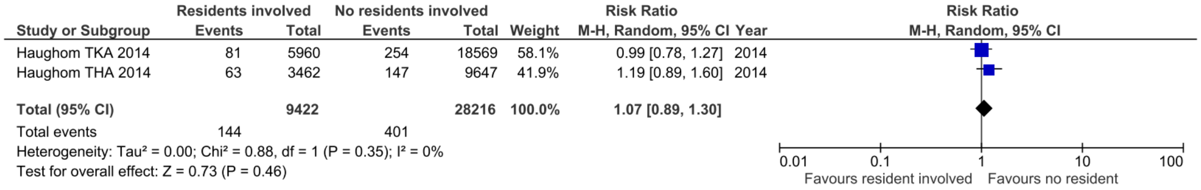

L: Unplanned re-admission*

Study or Subgroup	Residents involved Events	Residents involved Total	No residents involved Events	No residents involved Total	Weight	Risk Ratio M-H, Fixed, 95% CI
Haughom THA 2014	5	3462	24	9647	42.7%	0.58 [0.22, 1.52]
Haughom TKA 2014	14	5960	35	18569	57.3%	1.25 [0.67, 2.31]
**Total (95% CI)**		**9422**		**28216**	**100.0%**	**0.96 [0.57, 1.61]**
Total events	19		59			

Heterogeneity: Chi² = 1.73, df = 1 (P = 0.19); I² = 42%
Test for overall effect: Z = 0.15 (P = 0.88)

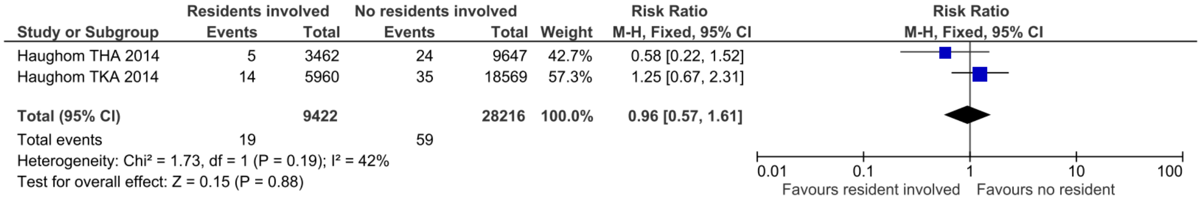

* The 4 NSQIP studies (13-16) had overlapping cohorts. Both studies of Haughom et al. were included since they also included the cohorts of Bohl et al. and Schoenfeld et al.
